# Spectrum-Effect Relationship between UPLC Fingerprints and Antilung Cancer Effect of Si Jun Zi Tang

**DOI:** 10.1155/2019/7282681

**Published:** 2019-09-22

**Authors:** Xiaowei Zhou, Ying Li, Mingyu Zhang, Junjie Hao, Qiong Gu, Haiyang Liu, Wei Chen, Yafei Shi, Bin Dong, Yuanyuan Zhang, Chunyu Li, Guohui Li

**Affiliations:** ^1^National Cancer Center/National Clinical Research Center for Cancer/Cancer Hospital, Chinese Academy of Medical Sciences and Peking Union Medical College, Beijing, China; ^2^School of Pharmacy, Chengdu University of Traditional Chinese Medicine, Chengdu, China; ^3^Tonghua Institute for Food and Drug Control, Tonghua, China

## Abstract

Nowadays, an increasing number of patients are seriously affected by lung cancer. Si Jun Zi Tang (SJZ), a four-herb Chinese medicine formula first described approximately one thousand years ago, is often prescribed for cancer patients as a complementary therapy. But the research on the effective materials for treating cancer using SJZ was rarely reported. To solve this problem, we evaluate the inhibitory effect of 10 samples of SJZ from different origins on PC9 cells. Ultraperformance liquid chromatography (UPLC) and hierarchical cluster analysis (HCA) along with canonical correlation analysis (CCA) and bioactivity validation were used to investigate the underlying correlation between the chemical ingredients and the antiproliferative effect of SJZ on PC9 cells. The evaluation indicated that 10 batches of SJZ could inhibit proliferation of PC9 cells and there was a notable difference in pharmacological activity between the different SJZ samples. The results of CCA and multivariate statistical analysis indicated that ginsenoside Ro and ginsenoside Rg1 might be active constituents of the antiproliferative effect as determined by spectrum-effect relationships. The results showed that bioassay and spectrum-effect relationships are suitable to associate sample quality with the active ingredient associated with clinical efficacy. And our finding would provide foundation and further understanding of the quality evaluation of traditional Chinese medicine decoction.

## 1. Introduction

Lung cancer is the leading cause of cancer-related mortality worldwide. 1.8 million people are diagnosed with lung cancer, and 1.6 million people die from this disease every year [1, 2]. Malignant proliferation of tumor cells is one of the leading causes of death. Traditional Chinese medicines (TCMs) have been used to treat a variety of tumor with the obvious advantages of fewer side effects [3, 4]. Si Jun Zi Tang (SJZ) was first described in the “Prescriptions People's Welfare Pharmacy” about a thousand years ago and consisted of *Panax ginseng* C.A.Mey., *Atractylodes macrocephala* Koidz., *Poria cocos* (Schw.) Wolf., and *Glycyrrhiza uralensis* Fisch. at a proportion of 3 : 3 : 3 : 2. As a representative formula for tonic TCM, SJZ has the effect of replenishing qi and strengthening spleen and is usually used to regulate gastrointestinal function, anti-inflammatory, and enhances immunity [5, 6]. Furthermore, it has been found that SJZ played a crucial role in preventing tumor recurrence and metastasis, improving quality of life, and prolonging the overall survival time, across a range of lung cancer, colorectal cancer, liver cancer, and gastric cancer [7–11]. Our previous research has shown that SJZ significantly suppresses Lewis lung cancer (LLC) growth in LLC-bearing mice [12]. However, the chemical composition of SJZ against lung cancer remains unclear, and it is difficult to control the quality linked to the clinical effects.

UPLC fingerprint is a useful tool for identifying authenticity, assessing quality, and distinguishing the ingredients of TCMs [13]. The spectrum-effect relationship that determines the correlations between fingerprint and biological activity is a scientific method for elucidating the pharmacodynamic basis and establishing a method for controlling the quality of TCMs [14–17].

Here, we evaluated the effect of SJZ on PC9 cells by a spectral-effect relationship approach. The IC50 of SJZ on PC9 cells was determined by analyzing ten batches of SJZ from different geographic regions. UPLC was performed to generate fingerprints of SJZ. Furthermore, UPLC and correlation analysis statistical method were used in combination to elucidate the relationship between pharmacological activity and chemical properties of 10 SJZ samples for characterizing their spectrum-effect relationships.

## 2. Materials and Methods

### 2.1. Chemicals, Reagents, and Materials

A total of 10 batches of SJZ comprising of *Panax ginseng* C.A.Mey. (R), *Atractylodes macrocephala* Koidz. (B), *Poria cocos* (Schw.) Wolf. (F), and *Glycyrrhiza uralensis* Fisch. (G) were included in the present study. Ten batches of *R* were purchased from China Medico Technology Co. Ltd. in Tianjin and designated as RS1–RS10. Ten batches of *B*, *F*, and *G* were obtained from various Chinese herbal medicine markets in several provinces of China, such as Heilongjiang, Jilin, Hunan, Shenyang, Guangxi, Beijing, and Sichuan. All the species were identified by Professor Xiao-he Xiao, a taxonomist at the China Military Institute of Chinese Medicine.

Phosphoric acid of HPLC grade was purchased from Beijing Chemical Works (Beijing, China). Acetonitrile of HPLC grade was bought from Fisher Scientific Co. (Fair Lawn, NJ, USA). Ultrapure distilled water was obtained from a Millipore Milli-Q-Plus system (Millipore, Bedford, MA, USA) [18]. The ginsenoside Rg1, Re, Rb1, Rc, Ro, and Rd, liquiritin, ammonium glycyrrhetate, and atractylenolide III were acquired from Chengdu Chroma-Biotechnology Co. Ltd. (Chengdu, China).

CCK-8 kit was purchased from Dojindo Laboratories (Kumamoto, Japan). DMEM high-sugar medium, fetal bovine serum, penicillin, and streptomycin were obtained from Gibco (Carlsbad, CA, USA).

PC9 cell line was purchased from the Fenghui Biotechnologies Inc. (Hunan, China), and it was authenticated by Hunan TSINGKE Biological Technology Co. Ltd.

### 2.2. Instruments

Waters Acquity UPLC system (Waters, Milford, MA, USA) consisted of a photodiode array (PDA) detector, column compartment, autosampler manager, and a binary solvent delivery pump and was connected to Waters Empower 2 software [18]. Enzyme microplate reader was purchased from BioTek, Winooski, 126 VT, USA.

### 2.3. Plant Sample Preparation

The plant material of *R*, *B*, *F*, and *G* was ground into powder using a mill. 2.2 g SJZ powder consisted of *R*, *B*, *F*, and *G* powder (ratio 3 : 3 : 3 : 2) and were dissolved in 20 mL distilled water. The mixture was vortexed and heated to boil and kept faint boiling for 1 h. The sample was cooled, centrifuged for ten minutes at 3000 rpm, and then concentrated by a rotary evaporator.

### 2.4. Preparation of Reference Standard Solution

Standard solutions were prepared by adding accurately weighed amounts of ginsenoside Rg1, Re, Rb1, Rc, Ro, and Rd, liquiritin, ammonium glycyrrhetate, and atractylenolide III dissolving with methanol to produce stock solution and stored at 4°C. Stock solutions of all the standards were diluted to the required concentrations and then mixed immediately before analyses. The final concentrations were 84.2 *μ*g/mL (Rg1), 96.8 *μ*g/mL (Re), 105.0 *μ*g/mL (Rb1), 86.4 *μ*g/mL (Rc), 95.0 *μ*g/mL (Ro), 210.0 *μ*g/mL (Rd), 372.0 *μ*g/mL (liquiritin), 398.0 *μ*g/mL (glycyrrhetate), and 30.8 *μ*g/mL (atractylenolide III), respectively.

### 2.5. UPLC Conditions

All the solutions were filtered through the 0.22 *μ*m microporous membrane before they were injected into the UPLC system. The chromatographic separation was performed using an ACQUITY UPLC BEH C_18_ column (2.1 mm × 100 mm i.d., 1.7 *μ*m particle size) (Water, Milford, MA, USA), operated at 30°C. The mobile phase was a mixture of solvent A (acetonitrile) and solvent B (0.1% formic acid in water). Gradient elution was developed under the following conditions: 0–10 min, 10–19% A; 10–16 min, 19%–21% A; 16–21 min, 21–32% A; 21–26 min, 32%–41% A; 26–30 min, 41–45% A; 30–35 min, 45–48% A; 35–36 min, 48–53% A; 36–40 min, 53–60% A; 40–45 min, 60–70% A; 45–55 min, 70–90% A; 55–60 min, 90–100% A; and 60–62 min, 100–10% A. The flow rate was kept constant at 0.25 mL·min^−1^. The effluent was monitored at 203 nm, and the sample injection volume was 5.0 *μ*L.

### 2.6. Cell and Cell Culture

PC9 cells were cultured in the DMEM high-sugar medium supplemented with 10% fetal bovine serum, penicillin (100 U/mL), and streptomycin (100 *μ*g/mL) at 37°C under a humidified atmosphere containing 5% CO_2_.

### 2.7. Cells Viability Assay

All ten water extracts of SJZ were dissolved in the 1 mL DMEM high-sugar medium. After incubation under 5% CO_2_ at 37°C for 24 h, PC9 cells (10^4^ cells per well) were subsequently inoculated onto 96-well plates and cultured for 24 h before onset of treatment. Then, the supernatant was discarded, and cells were treated with SJZ samples over a broad dose range to establish growth curves for 48 h. After that, cells were incubated for an additional 1 h with the CCK-8 reagent (100 *μ*L/mL medium). The absorbance was determined at 450 nm wavelength. The experiments were performed in triplicate. The proliferative inhibition rate was measured using the following formula:

(1) $$\begin{array}{c} \text{inhibition rate }\left(\text{\%}\right)=\frac{\left(A_{\text{control}}-A_{\text{sample}}\right)}{\left(A_{\text{control}}-A_{\text{blank}}\right)}\times 100\%. \end{array}$$

The IC50 (50% inhibitory concentration) value was calculated by nonlinear regression analysis using Graph-Pad Prism software (San Diego, CA, USA).

### 2.8. HCA

HCA is a multivariate analysis method that sorts specimens into clusters. This analysis can divide objects into specific clusters by means of maximizing homogeneity within each cluster while also maximizing heterogeneity between cluster [19, 20]. In this study, HCA was used to assess correlations in the UPLC fingerprints of 10 SJZ samples and was performed with Metabo Analyst 3.0 (http://www.metaboanalyst.ca/).

### 2.9. CCA

CCA is to study the correlation between two continuous variables, extracting the main principal components of them and finding their linear combinations of two sets of variables. Pearson's correlation coefficient is used to quantify the degree of colocalization between paired data. In our study, CCA was used to analyze the relevance between the peak area values from the UPLC fingerprints and IC50 from the measurement of inhibition on PC9 cells using SPSS statistics software (SPSS for Windows 17.0, SPSS Inc., USA).

## 3. Results

### 3.1. Effect of SJZ on PC9 Cell Viability

**Figure 1:**
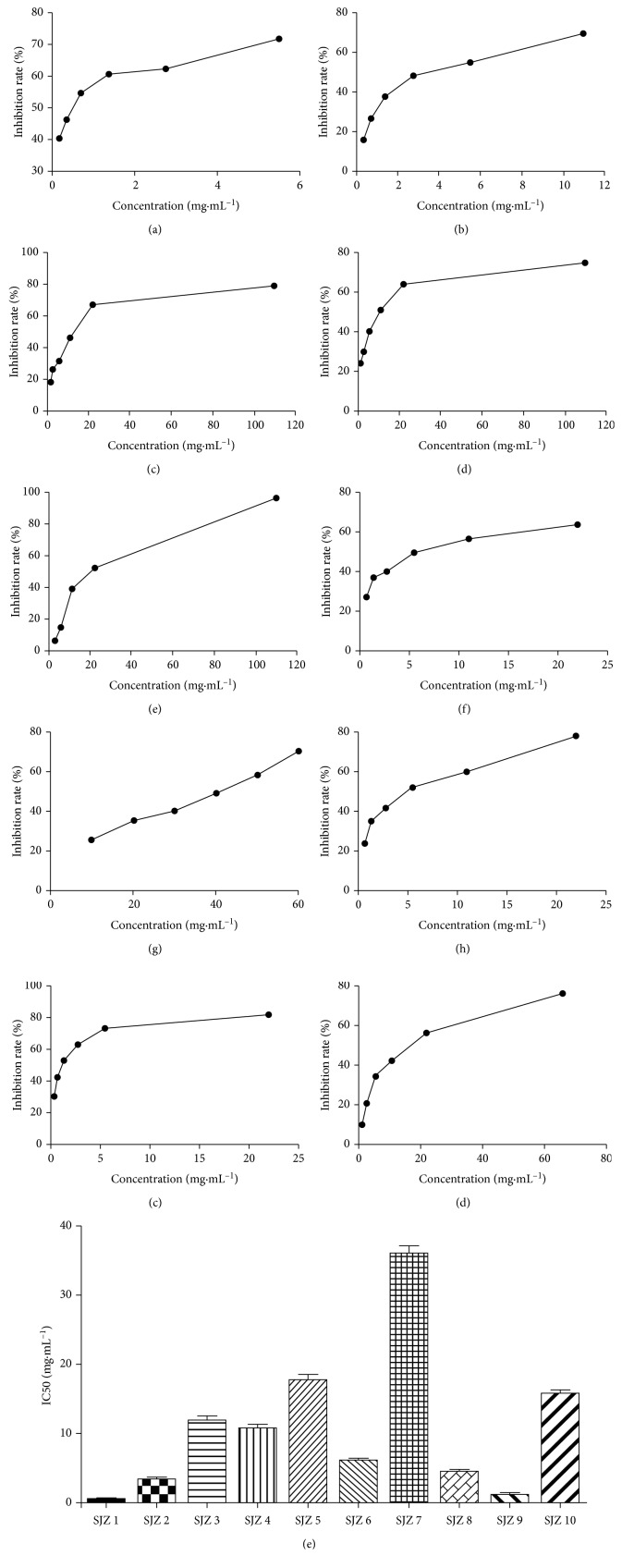
Inhibitory effects of ten batches of SJZ on PC9 cells. SJZ1-10 (a–j); IC50 of SJZ1-10 on PC9 cells (k).

CCK-8 assays were performed to investigate the effects of SJZ on the proliferation of PC9 cells. After treatment for 48 h with different concentrations, all ten batches of SJZ exhibited growth-inhibitory effects against PC9 cells which was dose-dependent (Figures 1(a)–1(j)). Values of IC50, the concentration of the extract required to inhibit cell growth by 50% of the control level, were estimated from the corresponding concentration and growth-inhibition plot. The bioactivities of samples showed various IC50 values for PC9 cells, from 0.48 to 35.95 mg/mL (Figure 1(k)). It seemed that the growth-inhibitory effect of the SJZ1 was strongest as it had the lowest IC50.

### 3.2. Analysis of UPLC Fingerprints

**Figure 2:**
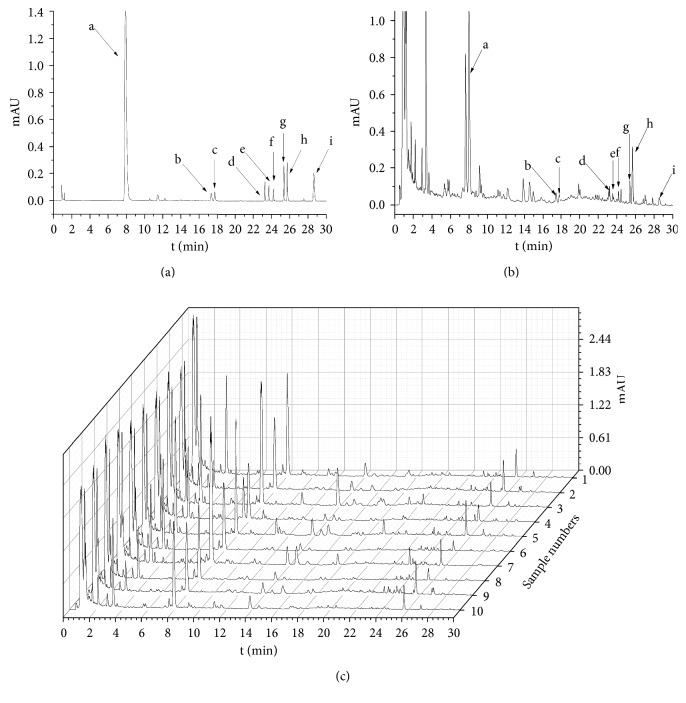
UPLC chromatogram of reference substances (a), test samples (b), and ten batches of SJZ (c). Nine peaks were identified by comparison with standard substances: (a) liquiritin; (b) ginsenoside Rg1, (c) Re, (d) Rb1, (e) Rc, (f) Ro, and (g) Rd; (h) ammonium glycyrrhetate; (i) atractylenolide III.

Ten SJZ samples were analyzed by UPLC–UV. The fingerprints of their UV chromatograms are shown in Figure 2. Under optimized conditions, chromatograms were generated for all batches of SJZ (Figure 2(c)) as well as for a mixture of reference substances (Figure 2(a)) and a test sample of SJZ4 (Figure 2(b)). The professional similarities were calculated using the Similarity Evaluation System for Chromatographic Fingerprint of Herbal Medicines (Version 2012.130723). Similarities between the reference fingerprint and each chromatographic profile of the ten batches of SJZ samples were evaluated by calculating the correlation coefficient. There were similar chemical profiles across different batches, and nine common peaks were found between 1 and 30 min time intervals by comparing ultraviolet spectra and UPLC retention times for the ten chromatograms. By comparing with the fingerprints of the reference substances, nine common peaks (a, b, c, d, e, f, g, h and i) were identified as liquiritin, ginsenoside Rg1, Re, Rb1, Rc, Ro, and Rd, ammonium glycyrrhetate, and atractylenolide III. And the highest peak in the fingerprints was peak a. As shown in Table 1, some differences were noted among data collected for the common peaks, such that the coefficients of variance (RSD%) for all common peaks were larger than 34.92%, which indicated that the content of chemical constituents was variant in different batches of SJZ from different origins.

### 3.3. Results of Hierarchical Clustering Analysis

**Figure 3:**
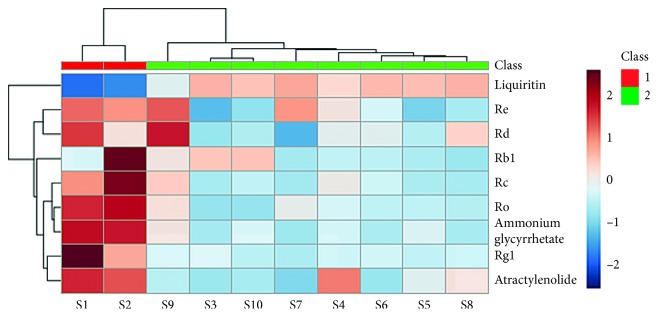
HCA heat map for ten SJZ samples and nine chemical compounds (right of the map).

A heat map is a graphical representation of data where each colored cell corresponds to an individual value contained in a matrix. Adjacent by HCA, the nine common peaks across ten SJZ samples were visualized by a heat map, and two clusters could be detected from Figure 3. Cluster 1 was composed of S1-S2, and cluster 2 consisted of S3–S10. In the heat map, the deeper color indicates the peak area of the SJZ samples of higher intensity. The results suggested that the content of chemical constituents contained in samples in cluster 1 was higher than that in cluster 2. The differences in the chemical ingredients could be related to the different geographic locations. The results showed that the SJZ samples from different origins had similar chemical fingerprint, and HCA could initially separate the samples at the chemical level.

### 3.4. Analysis of the Spectrum-Effect Relationship

**Figure 4:**
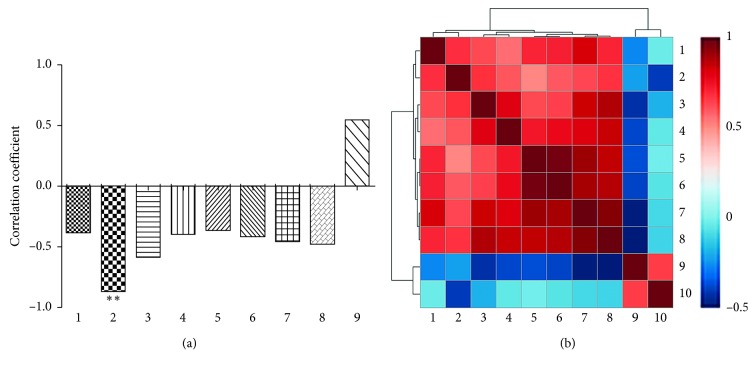
Analysis of the spectrum-effect relationship: (a) the correlation coefficient between the content of chemical compounds and bioactivity; (b) thermograph of the correlation analysis of all factors. (1) Ginsenoside Re; (2) ginsenoside Ro; (3) ginsenoside Rg1; (4) atractylenolide III; (5) ginsenoside Rc; (6) ginsenoside Rd; (7) ginsenoside Rb1; (8) ammonium glycyrrhetate; (9) liquiritin; (10) IC50. ^*∗∗*^*p* < 0.01.

CCA was used to study the spectrum-effect relationships between IC50 and area values of nine common peaks. As shown in Figure 4, the correlation coefficients demonstrated that eight peaks, namely, b, c, d, e, f, g, h, and i, were negatively correlated, while peak a was positively correlated. The value of IC50 which refers to the concentration to inhibit cell growth by 50% of the control level was negatively correlated with antitumor bioactivity of the SJZ sample. The results suggested that these compounds could inhibit the proliferation of PC9 cells. Eight correlated peaks, b, c, d, e, f, g, h, and i, have been identified previously as ginsenoside Rg1, Re, Rb1, Rc, Ro, and Rd, ammonium glycyrrhetate, and atractylenolide III, respectively. Ginsenoside Ro was the most correlated (*R* = −0.862) to the biological effect, and the bioactivity of ginsenoside Rg1 was the second highest (*R* = −0.576). Therefore, the above results indicate that ginsenoside Ro and ginsenoside Rg1 are the major antiproliferative constituents.

### 3.5. Experimental Validation

**Figure 5:**
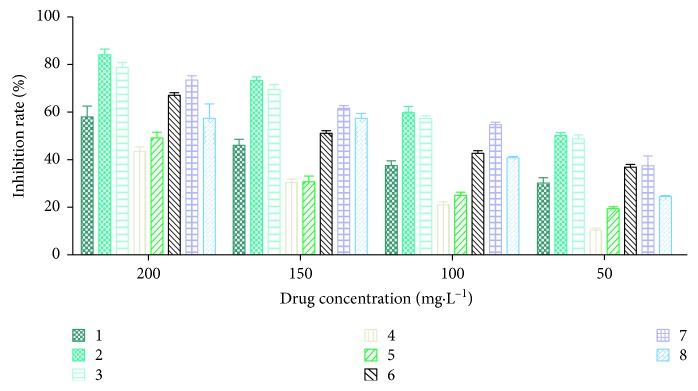
Dose-response curves of eight constituents inhibiting PC9 cell proliferation. (1) Ginsenoside Re; (2) ginsenoside Ro; (3) ginsenoside Rg1; (4) atractylenolide III; (5) ginsenoside Rc; (6) ginsenoside Rd; (7) ginsenoside Rb1; (8) ammonium glycyrrhetate.

Eight correlated peaks, ginsenoside Rg1, Re, Rb1, Rc, Ro, and Rd, ammonium glycyrrhetate, and atractylenolide III, were positively correlated with inhibition activity of PC9 cells. To validate the antiproliferation effect of eight constituents, the inhibition on PC9 cells of the eight compounds was determined at the same concentration. As shown in Figure 5, all samples could inhibit the proliferation of PC9 cells in a dose-dependent manner, and the inhibitory effect by ginsenoside Ro was highest than others, and ginsenoside Rg1 was the second highest, which is in accordance with the abovementioned results.

## 4. Discussion

TCM decoction is an oral liquid preparation prepared by decocting several Chinese herbal medicines according to the theory of “Jun-Chen-Zuo-Shi” and appropriate dosage ratio. It has the characteristics of good absorption, quick onset, and flexible composition, which fully embodies the essence of syndrome differentiation and treatment of TCM [21, 22]. Therefore, it is especially important to control the quality of TCM decoction because it directly affects clinical efficacy. However, due to the multitarget and multicomponent characteristics of TCM decoction, it is difficult to control the quality of TCM decoction and it has become a hot topic in the field of decoction research. Recently, many studies have proved that the spectral-effect relationship should be an effective method to control the quality of TCMs because it can connect chromatographic fingerprints with biological activity to explore quality markers related to clinical efficacy [23]. So, to ensure the quality and therapeutic consistency of TCM decoction, this approach may be a promising strategy to control its quality. The experimental results presented here indicate that ginsenoside Ro and ginsenoside Rg1 may be the main active components for inhibiting the proliferation of PC9 cells.

Consistent with this work, it has been reported that ginsenosides Ro and Rg1 could inhibit various types of cancer cells. Ginsenosides Ro could suppress autophagy by inhibiting autophagosome-lysosome fusion to sensitize esophageal cancer cells to 5-Fu-induced cell death [24] and inhibit the metastatic dissemination of colon cancer cells HT29 by suppressing the expression of *p*-ERK1/2 in HT29 cells [25]. Additionally, ginsenosides Ro is one of the bioactive constituents of ShenMai, which is an adjuvant therapy for cancer by inhibiting human organic anion-transporting polypeptides (OATP)1B [26]. Meanwhile, ginsenosides Rg1 could exert cytotoxic activity against human lung cancer cell lines A549, H1264, H1299, and Calu-6 [27] and suppress thyroid cancer proliferation and migration by enhancing the expression of Cx31 [28]. Moreover, ginsenosides Rg1 could sensitize hepatoblastoma cells to DNA-damaging agent, making ginsenosides Rg1 a promising chemotherapeutic agent [29], and it is also expected to become a preventive vaccine adjuvant for lymphoma by activating dendritic cells [30]. Thus, ginsenosides Rg1 and ginsenosides Ro may be used as new markers for quality control of SJZ treating lung cancer in the future.

Our findings suggested that spectrum-effect relationship should be a useful tool to associate the “quality marker” with clinical efficacy of TCM decoction, and it also provided a paradigm for the quality control of other anticancer TCM decoction. However, the two compounds cannot be applied as indicators of the overall quality control of SJZ because antiproliferative effect on PC9 cells is only one of its various clinical effects. Moreover, we mainly focused on compounds with a strong UV signal due to instrument limitations (PDA detector). Ginsenoside Rg3 played an important role in the prevention and treatment of cancer, but due to its low content, it could not be detected in this research [31, 32]. Therefore, further research is needed to determine whether ginsenosides Ro and Rg1 can be used as quality control markers for treating other diseases and whether there are other potential active components against lung cancer in SJZ. Next, to explore more active constituents or metabolites, mass spectrometry analysis will be considered for this research.

## Figures and Tables

**Table 1:** The relative peak area of nine common peaks measured by UPLC.

Sample	Peak area of each compound								
	a	b	c	d	e	f	g	h	i
S1	71	2499	548	344	606	839	480	2971	358
S2	683	1421	623	1306	1189	1124	384	3568	391
S3	25039	1769	741	2129	819	664	764	2523	293
S4	11207	809	818	602	792	682	591	2100	604
S5	20198	964	729	818	705	899	757	3657	508
S6	11626	772	638	531	569	507	535	1285	135
S7	30946	740	2396	1085	938	1821	622	2638	239
S8	12341	723	544	391	403	489	666	1262	361
S9	12421	1161	1597	1234	1363	1256	1457	3885	293
S10	13951	577	576	1281	612	392	516	2465	209
RSD%	69.65	52.53	65.53	56.45	36.79	50.13	44.11	34.92	41

RSD = *σ*/*μ* *∗* 100; RSD is the relative standard deviation; *σ* is the standard deviation; *μ* is the average value of peak area.

## Data Availability

The data used to support the findings of this study are available from the corresponding author upon request.
